# Cross-level interaction between individual socioeconomic status and regional deprivation on overall survival after onset of ischemic stroke: National health insurance cohort sample data from 2002 to 2013

**DOI:** 10.1016/j.je.2016.08.020

**Published:** 2017-07-05

**Authors:** Jaeyong Shin, Young Choi, Seung Woo Kim, Sang Gyu Lee, Eun-Cheol Park

**Affiliations:** a Department of Preventive Medicine, Yonsei University, College of Medicine, Seoul, South Korea; b Institute of Health Services Research, Yonsei University, College of Medicine, Seoul, South Korea; c Department of Public Health, Yonsei University Graduate School, Seoul, South Korea; d Department of Neurology, Yonsei University, College of Medicine, Seoul, South Korea; e Department of Hospital Management, Yonsei University Graduate School of Public Health, Seoul, South Korea

**Keywords:** Stroke, Mortality, Regional deprivation, Socioeconomic status

## Abstract

**Introduction:**

The literature on stroke mortality and neighborhood effect is characterized by studies that are often Western society-oriented, with a lack of racial and cultural diversity. We estimated the effect of cross-level interaction between individual and regional socioeconomic status on the survival after onset of ischemic stroke.

**Methods:**

We selected newly diagnosed ischemic stroke patients from 2002 to 2013 using stratified representative sampling data of 1,025,340 subjects. A total of 37,044 patients over the 10 years from 2004 to 2013 had newly diagnosed stroke. We calculated hazard ratios (HR) of 12- and 36-month mortality using the Cox proportional hazard model, with the reference group as stroke patients with high income in advantaged regions.

**Results:**

For the middle income level, the patients in advantaged regions showed low HRs for overall mortality (12-month HR 1.27; 95% confidence interval [CI], 1.13–1.44; 36-month HR 1.25; 95% CI, 1.14–1.37) compared to the others in disadvantaged regions (12-month HR 1.36; 95% CI, 1.19–1.56; 36-month HR 1.30; 95% CI, 1.17–1.44). Interestingly, for the low income level, the patients in advantaged regions showed high HRs for overall mortality (12-month HR 1.27; 95% CI, 1.13–1.44; 36-month HR 1.33; 95% CI, 1.22–1.46) compared to the others in disadvantaged regions (12-month HR 1.25; 95% CI, 1.09–1.43; 36-month HR 1.30; 95% CI, 1.18–1.44).

**Conclusion:**

Although we need to perform further investigations to determine the exact mechanisms, regional deprivation, as well as medical factors, might be associated with survival after onset of ischemic stroke in low-income patients.

## Highlights

• Regional socioeconomic status (SES) may differently affect mortality by income.
• Impact of area SES on morality among cerebrovascular patients was evaluated.
• Among middle-income patients, poor SES area is associated with poor mortality.
• Poor SES area is associated with better mortality among low-income patients.

## Introduction

Numerous studies have already demonstrated the relationship between socioeconomic status and health outcomes.^1^, ^2^, ^3^, ^4^ Stroke might be a preventable and curable disease if patients promptly arrive at well-equipped hospitals.^5^, ^6^, ^7^ Furthermore, risk factors for stroke can be reduced by controlling pre-existing chronic diseases, such as hypertension, dyslipidemia, and atrial fibrillation.^8^ Despite such improved medical circumstances, the stroke mortality rate remains high.^9^ In Korea, more than 230,000 years of healthy life are lost annually because of ischemic stroke, necessitating prompt action.^10^

Both individual and regional SES are independently associated with the incidence of stroke.^5^, ^6^, ^7^, ^11^, ^12^ However, the mechanisms whereby regional SES affects stroke incidence are not well understood.^13^, ^14^ Although regional deprivation is associated with stroke incidence, it remains unclear whether regional deprivation is truly related to the stroke mortality rate. Moreover, different types of medical coverage might encourage different uses of medical resources for prevention, treatment, and rehabilitation.

Although many developed countries, including Korea, are operating with a universal health coverage system, health disparities still exist between different socioeconomic groups. However, the association between stroke and SES has been demonstrated mostly in Western societies. The objective of this study was to examine effect of the cross-level interaction between individual and regional SES on the survival after onset of ischemic stroke in the Korean National Health Insurance Claim Database (NHICD).

## Methods

### Data

This study used Korean National Health Insurance (NHI) cohort data, which include information about approximately 1 million patients; the data are random-sample and stratified according to age, sex, region, health insurance type, income decile, and individual total medical costs from 2002 to 2013. All Korean citizens are obligated to join the National Health Security System, which comprises the NHI and Medical Aid and is overseen by the Ministry of Health and Welfare. The data include unique anonymous numbers for each patient, including age, sex, type of insurance, a list of diagnoses according to the International Classification of Diseases version 10 (ICD-10), medical costs claimed, prescribed drugs, and medical history. In addition, unique anonymous numbers are linked to information on mortality obtained from the Korean National Statistical Office. The institutional review board (IRB) of the Graduate School of Public Health in Yonsei University approved this study in 2014 (IRB approval number: 2-10409390-AB-N-01-2014-239).

### Participants

We conducted a cohort study of patients newly diagnosed with ischemic stroke (ICD-10 code: *I.63*), using a 2.5% stratified random sample (n = 1,025,340) of all citizens on December 31, 2002 (eFig. 1 and eFig. 2). From this pool, we selected 44,769 patients with a primary diagnosis of ischemic stroke in the 12 years from 2002 to 2013. Among them, 7511 patients with pre-existing ischemic stroke in 2002 and 2003 were removed; after these patients were eliminated, 37,258 stroke patients who were free of such disease prior to 2002 and 2003 were selected. Since the regional deprivation in the study reflected the economic situation in 2005, we also excluded 214 ischemic stroke patients who lived in newly created municipals after 2005. Finally, 37,044 patients were enrolled in the study. Among the enrolled participants, 856 patients were lost to follow-up; these participants were censored at the end of the last year with an available medical record.

### Covariates

Demographic characteristics, including age, sex, and area of residence were analyzed, as well as medical history of hypertension, diabetes, hyperlipidemia, atrial fibrillation, and ischemic heart disease. If the patients lived in Seoul, which is the capital of Korea, then we defined the living area as ‘capital’. Others living in six metropolitan areas in Korea, including Busan, Gwangju, Ulsan, Incheon, Daejeon, and Daegu, were included in ‘metropolitan’ category. Otherwise, the patients were defined as ‘rural’.

In addition, we adjusted for the severity of stroke using the length of intensive care unit (ICU) stay during the first year after ischemic stroke onset and presence of cerebral disability based on the Korean version of the modified Barthel index.^15^, ^16^

We also considered the volume performance of hospitals where patients were initially diagnosed according to the number of beds. In addition, we divided all hospitals using a hierarchy with three components: general hospitals, hospitals, and clinics. The availability of computed tomography (CT) and magnetic resonance imaging (MRI) was included in analysis.

### Individual-level income measures

Regarding individual income levels, the NHI premium was used as a proxy measure of precise income because it is proportional to monthly income, including earnings and capital gains. In Korea, the type of health insurance is classified as national health insurance or medical aid. People can qualify for medical aid if their single-family household income is less than $600 per month; otherwise, they have national health insurance. People who have national health insurance based on employment pay a monthly insurance premium according to their annual salary, and people who are self-employed pay for their premium based on the value of their property. People who qualified for national health insurance were distributed from the 1st to the 100th percentile, and people who had medical aid were classified as 0 percentile. We categorized individual household incomes into three groups (Low, 0-30th percentile; Middle, 31st-70th percentile; High, 71st-100th percentile).

### Regional-level socioeconomic status

A summary measure was used to characterize regional-level deprivation. We used a modified Carstairs index with census data from 2005.^17^, ^18^ In previous studies,^18^, ^19^, ^20^ four variables from census data were used to calculate the Carstairs index: 1) residents in households headed by unskilled individuals, 2) unemployed males, 3) residents overcrowded, and 4) residents without a car.

However, because we could not obtain car ownership information from census data, we replaced ‘residents without a car’ with ‘residences not owner occupied.’ The values were derived for each area using 2% microdata from the 2005 Population and Housing Census from the Korea National Statistical Office. A positive, higher score on the index denotes greater deprivation. This modified index displays a significant association with health and has been shown to be robust for consistency over time and over outcome variables in many previous studies in Korea.^21^ Thus, the use of this verified area deprivation index provides more reliable results and could help in developing an area-deprived index suitable for health-related studies in South Korea.^22^

The regional deprivation index was calculated at the municipal level of Si (city), Gun (county), and Gu (borough) by merging these four basic indicators, similar to the method used to calculate the Carstairs index. The municipal areas are geographical units covering all small areas in Korea. In Korea, there were 182,948 citizens and 63,549 households per unit in 2005. Moreover, the number of beds and hospitals in disadvantaged areas were 7.62 beds per 1000 populations and 14.7 hospitals per 10,000 populations, while corresponding rates in advantaged areas were 8.6 beds per 1000 population and 15.2 hospitals per 10,000 populations. We calculated z-scores at the level of Si, Gun, and Gu using the mean and standard deviation (SD) of the four indicators.

The z-score was calculated by subtracting the mean from the observed value for each indicator, dividing the standard deviation by this value, and then summing the four standardized z-scores. The indexes were categorized into two groups, advantaged and disadvantaged regions, based on the median value of the Carstairs index for regional characteristics, with advantaged regions having values less than or equal to the median, and disadvantaged regions having values greater than the median.

### Statistical analysis

Before statistical analyses, we assessed the distribution of the demographic characteristics among stroke patients at baseline. Continuous variables were expressed as means and SDs or medians and were compared using *t*-tests or the Kruskal–Wallis test, where appropriate. Baseline categorical variables were expressed as numbers and percentages, and were compared using the *χ*^2^ test.

We also estimated the adjusted hazard ratios (HRs) and 95% confidence intervals (CIs) by applying a Cox proportional-hazard regression model using a shared frailty model that allows individuals to be nested within regions and the intercept to vary among regions.^23^ In this model, we adjusted the individual, hospital, and regional characteristics as follows (individual factors: sex, residential area, medical history of hypertension, diabetes, hyperlipidemia, atrial fibrillation, and ischemic heart disease, length of ICU stay; hospital factors: presence of CT and MRI, the number of beds, the number of doctors, the hospital level; regional factor: Carstair index). The essential assumption of the proportional hazard model was satisfied by graphical proof. Mortality for ischemic stroke, stratified by individual income level and regional SES, was measured from the time of diagnosis, using overall survival after the ischemic stroke onset as the event variable. Cumulative 12-month and 36-month mortality rates and mortality curves were constructed and compared using the log-rank test. Model fitting was performed using the PROC LIFEREG command in SAS version 9.3 (SAS Institute Inc., Cary, NC, USA) and the gamma distribution was chosen.

## Results

### Demographic data and clinical characteristics

**Fig. 1:**
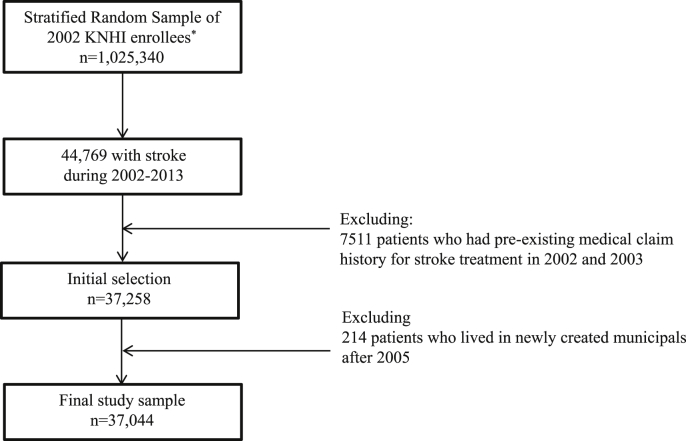
Study flow diagram.

We observed a total number of 37,044 participants with 33,269 person-years for 12-month mortality and 86,114 person-years for 36-month mortality (Table 1, eTable 1, and Fig. 1). There were 9.5 mortality cases per 100 person-years at 12 months and 6.3% per 100 person-years at 36 month (eTable 1).

**Table 1:** Demographic characteristics of individuals and neighborhoods for each mortality by follow-up period.

	12-month mortality				36-month mortality			
	Total	Deaths		p-value	Total	Deaths		p-value
		n	%			n	%	
**Individual income - neighborhood deprivation**								
High in advantaged	5472	422	7.7	0.001	4017	600	14.9	<0.001
High in disadvantaged	4929	391	7.9		3720	542	14.6	
Middle in advantaged	3544	284	8.0		2620	416	15.9	
Middle in disadvantaged	3717	266	7.2		2721	367	13.5	
Low in advantaged	2788	274	9.8		1836	344	18.7	
Low in disadvantaged	2927	268	9.2		1894	346	18.3	
**Individual characteristics**								
**Age group, years**								
≤39	642	1	0.2	<0.001	471	11	2.3	<0.001
40–49	1986	38	1.9		1495	52	3.5	
50–59	3999	96	2.4		2919	136	4.7	
60–69	7093	313	4.4		5382	527	9.8	
70–79	6811	690	10.1		4744	1074	22.6	
≥80	2828	757	26.8		1797	815	45.4	
**Sex**								
Male	11,112	905	8.1	0.980	7983	1314	16.5	0.002
Female	12,265	1000	8.2		8825	1301	14.7	
**Residential area**								
Rural	8435	780	9.2	<0.001	6282	1104	17.6	<0.001
Urban	14,942	1125	7.5		10,526	1511	14.4	
**Hypertension**								
No	7537	584	7.7	0.123	5551	757	13.6	<0.001
Yes	15,840	1321	8.3		11,257	1858	16.5	
**Diabetes**								
No	15,859	1292	8.1	0.986	11,690	1736	14.9	<0.001
Yes	7518	613	8.2		5118	879	17.2	
**Hypercholesterolemia**								
No	17,625	1606	9.1	<0.001	13,256	2248	17.0	<0.001
Yes	5752	299	5.2		3552	367	10.3	
**Atrial fibrillation**								
No	22,544	1737	7.7	<0.001	16,278	2443	15.0	<0.001
Yes	833	168	20.2		530	172	32.5	
**Ischemic Heart Disease**								
No	18,648	1469	7.9	0.003	13,678	2072	15.1	0.002
Yes	4729	436	9.2		3130	543	17.3	
**Disabled due to cerebral lesions**								
Healthy	21,748	1744	8.0	0.001	15,734	2371	15.1	<0.001
Mild	571	43	7.5		381	62	16.3	
Severe	1058	118	11.2		693	182	26.3	
**Length of stay in ICU, days**	Mean (SD)	Mean (SD)			Mean (SD)	Mean (SD)		
	1.34 (7.49)	5.76 (15.49)		<0.001	1.34 (7.54)	4.56 (14.59)		<0.001
**Hospital characteristics**								
**CT**								
No	2856	250	8.8	0.208	1813	299	16.5	0.245
Yes	20,521	1655	8.1		14,995	2316	15.5	
**MRI**								
No	4951	474	9.6	<0.001	3394	612	18.0	<0.001
Yes	18,426	1431	7.8		13,414	2003	14.9	
**Hierarchy of hospitals**								
General hospital	15,694	1195	7.6	<0.001	11,495	1670	14.5	<0.001
Hospital	3634	494	13.6		2453	597	24.3	
Clinics	4049	216	5.3		2860	348	12.2	
**Number of Beds/1000**	Mean (SD)	Mean (SD)			Mean (SD)	Mean (SD)		
	0.58 (0.51)	0.54 (0.51)			0.59 (0.51)	0.55 (0.51)		
**Community characteristics**								
	Mean (SD)	Mean (SD)			Mean (SD)	Mean (SD)		
**Smoking rate**	24.77 (1.83)	24.84 (1.72)		0.066	24.76 (1.81)	24.85 (1.75)		0.020
**Binge alcohol drinking rate**	16.61 (2.55)	16.56 (2.55)		0.344	16.62 (2.55)	16.55 (2.58)		0.232
**Moderate exercise rate**	21.70 (5.64)	22.16 (6.17)		0.001	21.77 (5.72)	22.27 (6.14)		<0.001
**Total**	23,377	1905	8.1		16,808	2615	15.6	

CT, computed tomography; ICU, intensive care unit; MRI, magnetic resonance imaging; SD, standard deviation.

Neighborhood socioeconomic status was based on the Carstairs index, standardized and averaged to form an index and divided into advantaged (below 50%) and disadvantaged (50% and above) neighborhoods.

A total of 17,418 men (47.0%) and 19,626 women (53.0%) were enrolled in this study.

The number of high-, middle-, and low-income ishemic stroke patients were 15,760 (42.5%), 11,102 (30.0%), and 10,182 (27.5%), respectively. In addition, there were 18,552 ischemic stroke patients (50.1%) in advantaged and 18,492 patients (49.9%) in disadvantaged regions. The other demographic characteristics are shown in Table 1.

### Univariate analysis

**Fig. 2:**
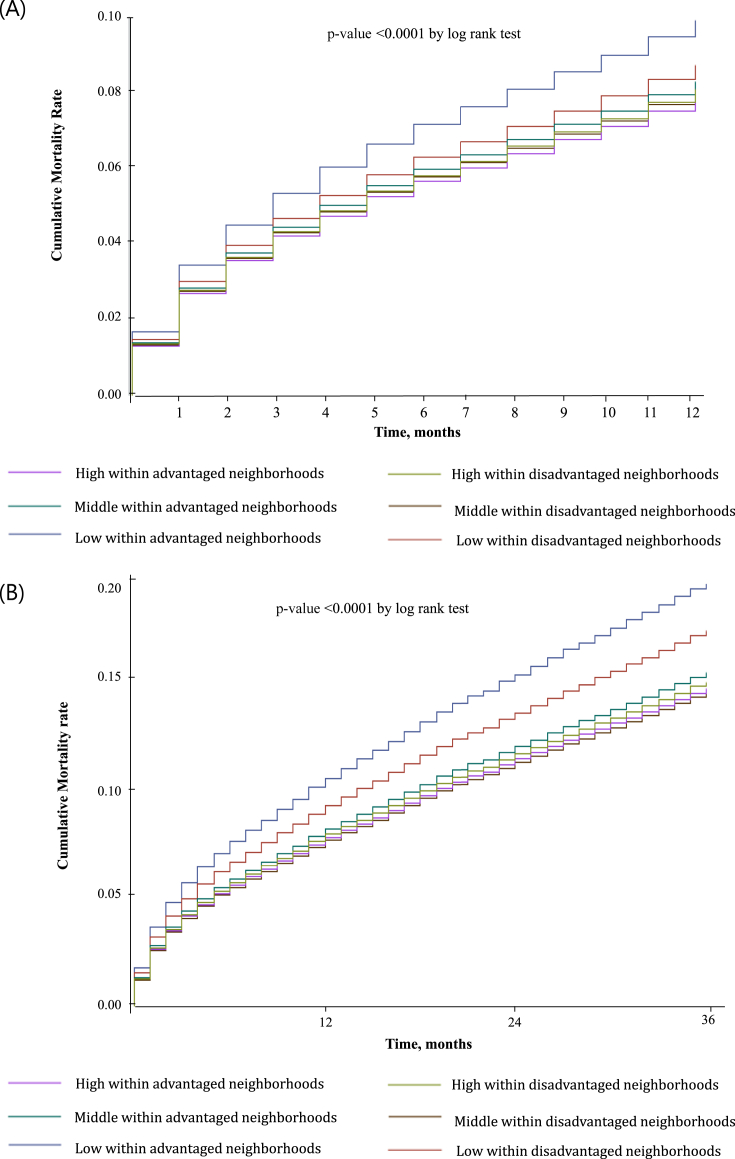
A. Cumulative mortality rates, 12 month. B. Cumulative mortality rates, 36 month.

Analysis of the combined effect of individual income and regional SES revealed that mortality rates were highest among stroke patients with low individual incomes residing in either advantaged or disadvantaged regions. Both the 12-month and 36-month cumulative mortality rates were highest among stroke patients who had low individual incomes and resided in advantaged regions (p < 0.001) (Fig. 2).

### Multivariate analysis according to 12- and 36-month mortality

Before the examination for the combined variable between individual income and regional deprivation, we performed the analysis for the separate association each and interaction (eTable 2). In terms of individual income, the low- (HR 1.34; 95% CI, 1.03–1.76) and middle-income patients (HR 1.34; 95% CI, 1.15–1.57) showed statistically higher adjusted HRs for all-cause mortality than the high-income patients. However, there was no statistical difference in regional deprivation and interaction. Interestingly, the sub-population in rural areas showed a statistical difference in the interaction term.

The results of the Cox proportional hazards model for 12- and 36-month mortality are shown in Table 2. In terms of the combined interesting variable, we set the group of high income in advantaged regions as the reference category.

**Table 2:** The adjusted hazard ratios of mortality for different follow-up periods.

	12-month mortality			36-month mortality		
	HR	95% CI	p-value	HR	95% CI	p-value
**Individual income - neighborhood deprivation**						
High in advantaged	1.00			1.00		
High in disadvantaged	1.00	0.86–1.15	0.971	0.91	0.80–1.04	0.156
Middle in advantaged	1.19	1.02–1.39	0.025	1.24	1.09–1.40	0.001
Middle in disadvantaged	1.09	0.93–1.28	0.306	1.09	0.95–1.26	0.220
Low in advantaged	1.25	1.07–1.46	0.005	1.30	1.14–1.49	<0.001
Low in disadvantaged	1.19	1.01–1.39	0.036	1.35	1.17–1.55	<0.001
**Individual characteristics**						
**Age group, years**						
≤39	1.00			1.00		
40–49	1.22	0.62–2.39	0.563	1.48	0.77–2.83	0.242
50–59	1.58	0.84–2.96	0.154	2.02	1.09–3.75	0.025
60–69	3.01	1.64–5.51	<0.001	4.41	2.42–8.02	<0.001
70–79	6.85	3.75–12.50	<0.001	10.92	6.01–19.83	<0.001
≥80	18.80	10.30–34.30	<0.001	26.13	14.37–47.51	<0.001
**Sex**						
Men	1.00			1.00		
Women	0.80	0.73–0.88	<0.001	0.73	0.67–0.79	<0.001
**Residential area**						
Rural	1.00			1.00		
Urban	1.06	0.95–1.19	0.314	1.03	0.93–1.15	0.582
**Hypertension**						
No	1.00			1.00		
Yes	0.81	0.73–0.90	<0.001	0.93	0.85–1.01	0.087
**Diabetes**						
No	1.00			1.00		
Yes	1.06	0.96–1.17	0.250	1.18	1.09–1.29	<0.001
**Hypercholesterolemia**						
No	1.00			1.00		
Yes	0.69	0.61–0.78	<0.001	0.68	0.61–0.76	<0.001
**Atrial fibrillation**						
No	1.00			1.00		
Yes	2.01	1.71–2.37	<0.001	1.75	1.49–2.05	<0.001
**Ischemic Heart Diseases**						
No	1.00			1.00		
Yes	1.10	0.98–1.23	0.112	1.05	0.95–1.16	0.321
**Disabled due to cerebral lesions**						
Healthy	1.00			1.00		
Mild	1.07	0.79–1.45	0.661	1.20	0.93–1.55	0.163
Severe	1.20	0.99–1.45	0.061	1.69	1.45–1.97	<0.001
**Length of stay in ICU, days**	1.02	1.02–1.02	<0.001	1.02	1.02–1.02	<0.001
**Hospital characteristics**						
**CT**		–			–	
No	1.00	–		1.00	–	
Yes	0.82	0.68–0.99	0.035	0.76	0.64–0.91	0.002
**MRI**		–			–	
No	1.00	–		1.00	–	
Yes	0.83	0.70–0.99	0.036	0.85	0.73–0.99	0.034
**Hierarchy of hospital**		–			–	
General hospital	1.00	–		1.00	–	
Hospital	1.24	1.07–1.45	0.005	1.27	1.11–1.44	0.001
Clinics	0.55	0.44–0.69	<0.001	0.61	0.50–0.74	<0.001
**Number of Beds/1000** **at the initial hospital**	1.05	0.92–1.19	0.486	1.01	0.91–1.13	0.801
**Community characteristics**						
**Smoking rate**	1.02	0.99–1.05	0.145	1.02	1.00–1.05	0.089
**Binge alcohol drinking rate**	1.00	0.98–1.02	0.839	1.00	0.98–1.01	0.625
**Moderate exercise rate**	1.00	0.99–1.01	0.984	1.00	0.99–1.01	0.829

CI, confidence interval; CT, computed tomography; HR, hazard ratio; ICU, intensive care unit; MRI, magnetic resonance imaging.

Note: Neighborhood SES was based on the Carstairs index, standardized and averaged to form an index and divided into advantaged (below 50%) and disadvantaged (50% and above) neighborhoods.

For the middle income level, the patients in advantaged regions showed low HRs for overall mortality (12-month HR 1.27; 95% CI, 1.13–1.44; 36-month HR 1.25; 95% CI, 1.14–1.37) compared to the others in disadvantaged regions (12-month HR 1.36; 95% CI, 1.19–1.56; 36-month HR 1.30; 95% CI, 1.17–1.44). Interestingly, for the low income level, the patients in advantaged regions showed high HRs for overall mortality (12-month HR 1.27; 95% CI, 1.13–1.44; 36-month HR 1.33; 95% CI, 1.22–1.46) compared to the others in disadvantaged regions (12-month HR 1.25; 95% CI, 1.09–1.43; 36-month HR 1.30; 95% CI, 1.18–1.44).

Regarding age, patients aged 80 years or older had the highest adjusted HRs for mortality at both 12 (HR 23.97; 95% CI, 14.11–40.72) and 36 months (HR 33.48; 95% CI, 21.01–53.36). Men also had higher HRs for 12- and 36-month mortality than women. Pre-existing atrial fibrillation was significantly associated with the risk of mortality at 12 and 36 months, while diabetes was significantly associated with the risk of mortality at 36 months only. An ICU stay longer than 1 day was significantly associated with mortality, regardless of the follow-up duration (12- and 36-month HR 1.03; 95% CI, 1.03–1.03).

In terms of hospital characteristics, the availability of MRI was associated with significantly lower mortality at 12 months and 36 months (12-month HR 0.69; 95% CI, 0.60–0.80; 36-month HR 0.76; 95% CI, 0.68–0.85).

Since differences in health resources between urban and rural settings might lead to different outcomes among stroke patients, we also performed subgroup analysis by residential area (Table 3). Among the ischemic stroke patients in the capital and metropolitan areas, the synergetic effect between individual income and regional deprivation was observed in both middle- and low-income groups. However, in rural areas, there was distinct paradoxical regional deprivation in low-income patients within advantaged regions.

**Table 3:** The adjusted hazard ratios for mortality by residential area.

Individual income - neighborhood deprivation	Urban			Rural		
	HR	95% CI	p-value	HR	95% CI	p-value
**12-month mortality**						
High in advantaged	1.00			1.00		
High in disadvantaged	0.97	0.81–1.16	0.750	1.04	0.81–1.33	0.762
Middle in advantaged	1.17	0.97–1.41	0.108	1.21	0.92–1.57	0.168
Middle in disadvantaged	1.11	0.91–1.36	0.296	1.02	0.77–1.35	0.914
Low in advantaged	1.28	1.05–1.55	0.014	1.21	0.93–1.58	0.161
Low in disadvantaged	1.08	0.88–1.33	0.454	1.35	1.04–1.77	0.027
**36-month mortality**						
High in advantaged	1.00			1.00		
High in disadvantaged	0.95	0.81–1.12	0.548	0.84	0.68–1.04	0.112
Middle in advantaged	1.31	1.12–1.53	0.001	1.11	0.89–1.39	0.362
Middle in disadvantaged	1.14	0.95–1.36	0.158	1.01	0.80–1.28	0.908
Low in advantaged	1.28	1.08–1.53	0.005	1.32	1.06–1.65	0.014
Low in disadvantaged	1.38	1.15–1.65	<0.001	1.30	1.02–1.65	0.032

CI, confidence interval; CT, computed tomography; HR, hazard ratio; ICU, intensive care unit; MRI, magnetic resonance imaging.

Note: Neighborhood socioeconomic status was based on the Carstairs index, standardized and averaged to form an index and divided into advantaged (below 50%) and disadvantaged (50% and above) neighborhoods.

## Discussion

This study examined the combined effect of individual income and regional SES on the risk of all-cause mortality among ischemic stroke patients under the national health insurance system in Korea. Stroke patients with low individual income had significantly greater all-cause mortality, after adjustment for a variety of comorbidities. Our findings are consistent with the results of studies investigating the relationship between individual SES and post-stroke survival in other populations.^24^ Several mechanisms might explain the higher mortality rate in low-income stroke patients. For instance, they might have a greater number of comorbidities and cerebrovascular risk factors, such as uncontrolled hypertension and diabetes.^2^, ^24^, ^25^ Moreover, reduced access to medical resources may contribute to their worse outcomes.^26^, ^27^, ^28^

Interestingly, we found higher mortality among stroke patients who had low individual incomes and lived in high-SES regions. This suggests that individuals with low incomes do not benefit appropriately from the higher quality of resources and knowledge generally available in advantaged regions.

Our results differed from the outcomes of some previous studies. According to one of the most recent studies, a higher region deprivation level was associated with a higher stroke incidence in Japan,^14^ although this study did not adjust for individual income. In another study from the United States, a higher risk of ischemic stroke incidence and mortality was observed in the most disadvantaged neighbourhoods.^11^, ^13^ However, it remains controversial whether the stroke mortality rate is truly worse in disadvantaged regions. In fact, there are many other studies indicating that adults with low individual SES in advantaged regions might have a higher mortality risk because of their regional deprivation and/or low relative social standing.^29^, ^30^

In terms of cardiovascular disease, several studies from the United States and Canada have been performed to examine the effect of the cross-level interaction between individual and regional SES on mortality.^31^, ^32^, ^33^, ^34^ Among them, three studies showed results in the same direction as ours, while the other showed no significant cross-level effects.^32^, ^33^

Although we do not know the exact mechanisms for the result, we suggest that there are several possible explanations, including regional deprivation and relative standing. Regarding the former, regional deprivation hinders stroke patients with low individual incomes in advantaged regions from using essential medical uses due to economic burden. The higher basic living expenses in advantaged regions might reduce disposable income and require individuals to work longer. Moreover, such people may live far away from health services and other essential services, compared with people with low individual incomes in disadvantaged regions. Ultimately, they are not able to use appropriate health resources because of financial burdens, lack of time, and geographical inaccessibility.

Regarding the other hypothesis of relative standing, a low relative standing in their communities might be related to the higher mortality among stroke patients. This hypothesis suggests that the disparity in one's social position relative to others in one's community influences one's mortality risk.^29^, ^35^ A relatively low SES might be related to limited resources to deal with stress, low social confidence, and lack of social support.^36^ All of these may induce tangible social discrimination and psychological stress.^37^, ^38^, ^39^, ^40^ This hypothesis is consistent with previous reports that a number of psychological and social factors have some influence on health via direct pathways involving biological mechanisms and via indirect pathways involving health behaviors.^39^

In addition, we suggest that the regional effect should be interpreted in one's own culture and circumstance of society. Our results differ from those in Western society. However, since one Taiwanese study reported a trend very similar to ours, we believe that some sociologic and cultural factors may influence important survival factors among stroke patients.^22^ In this study, Taiwanese acute myocardial infarction patients with low individual SES had an increased risk of death than those with high individual SES who resided in advantaged neighborhoods. Moreover, the low-income AMI patients in advantaged regions showed higher HR than the low-income patients in disadvantaged regions. For example, regional cohesion is associated with the stroke mortality rate. Thus, if one community in a disadvantaged region has stronger neighborhood cohesion than others, there might be a plausible explanation for these kinds of external effects of sociologic factors on the stroke mortality rate.

Regarding the medical aspects, a history of hypertension or hypercholesterolemia was associated with a lower risk of mortality. Older age, male sex, atrial fibrillation, and severe neurologic deficits have been suggested to be related to increased mortality in previous studies, as in ours. However, hypertension and hypercholesterolemia predicted a lower risk of mortality in the present study, whereas these factors were not significantly associated with mortality in previous studies.^41^, ^42^, ^43^, ^44^ This discrepancy may result from the lack of data on the etiology of ischemic stroke. Cardioembolic stroke is associated with high mortality, whereas stroke from small vessel disease is associated with low mortality.^45^, ^46^ Of the stroke subtypes, the prevalence of hypertension and hypercholesterolemia was relatively higher in stroke patients with small vessel disease. Thus, the association between hypertension and hypercholesterolemia and lower mortality in this study may have resulted from this medical use and prevention.

Before drawing conclusions, there are some limitations of this study that should be mentioned. First, there is a chance that other important covariates were omitted, such as education level, amount of regular exercise, and family structure. Although such factors are strongly associated with stroke mortality, they were not included because of the limited information available in the claim data. Second, we could not measure stroke severity. We tried to account for stroke severity using length of stay in the ICU and the cerebral disability grading using the modified Bethel index. In spite of these efforts, it was not possible to use other reliable stroke scales, such as the United States' National Institute of Health stroke scale and the Canadian neurologic scale.^47^, ^48^ Third, survival was used as the outcome variable, meaning we could not adjust for quality of life. Some survivors with severe stroke sequelae might experience an extremely poor quality of life. However, we could not obtain information regarding quality of life for all enrolled patients.

Nevertheless, the present findings are valuable because of the following strengths of our study. First, we used stratified random sampling from a national-level dataset that included more than 1,000,000 patients, thus securing the external validity of the study. Second, as this study was an observational cohort study, the association between the independent variables and survival is more confirmative than in a cross-sectional study. Third, we used survival and medical history data from national statistics and the NHI, which are the most accurate survival and disease databases in South Korea; therefore, the data about previous medical conditions and mortality were highly reliable.

## Conclusion

Among stroke patients with low individual incomes, the patients in advantaged regions had higher mortality rates than those in disadvantaged regions. Although we need to perform further investigations to determine the exact mechanisms, it seems that social and cultural factors, as well as medical and physiologic ones, may play a key role in stroke mortality.

## Conflicts of interest

None declared.
