# Supplementary material for: Cross-level interaction between individual socioeconomic status and regional deprivation on overall survival after onset of ischemic stroke: National health insurance cohort sample data from 2002 to 2013
Source: J Epidemiol. 2017 Jul 5;27(8):381–8. doi: 10.1016/j.je.2016.08.020 (PMC5549246; doi:10.1016/j.je.2016.08.020)

eTable 1. Person-years based on the characteristics of individuals and hopsitals

	12-month mortality					36-month mortality				
	Number of deaths	Person-years	Overall mortality per 100 PY			Number of deaths	Person-years	Overall mortality per 100 PY		
			Value	95% CI	95% CI			Value	95% CI	95% CI
**Individual income - Neighbourhood deprivation**										
High income in advantaged neighbourhood, 5	663	7458	8.9	8.2	9.6	1131	19,443	5.8	5.5	6.2
High income in disadvantaged neighbourhood, 6	619	6776	9.1	8.4	9.9	1049	17,650	5.9	5.6	6.3
Middle income in advantaged neighbourhood, 3	457	4861	9.4	8.6	10.3	779	12,714	6.1	5.7	6.6
Middle income in disadvantaged neighbourhood, 4	467	5152	9.1	8.3	9.9	775	13,597	5.7	5.3	6.1
Low income in advantaged neighbourhood, 1	490	4303	11.4	10.4	12.4	876	10,827	8.1	7.6	8.6
Low income in disadvantaged neighbourhood, 2	470	4720	10.0	9.1	10.9	841	11,882	7.1	6.6	7.6
**Individual characteristics**										
**Age group, years**										
<40	14	980	1.4	0.8	2.4	18	2693	0.7	0.4	1.1
40-49	55	2739	2.0	1.5	2.6	91	7540	1.2	1.0	1.5
50-59	152	5997	2.5	2.2	3.0	288	16,053	1.8	1.6	2.0
60-69	471	9423	5.0	4.6	5.5	887	25,287	3.5	3.3	3.7
70-79	1127	9893	11.4	10.7	12.1	2055	24,933	8.2	7.9	8.6
≥80	1347	4237	31.8	30.1	33.5	2112	9607	22.0	21.1	22.9
**Sex**										
Male	1540	15,586	9.9	9.4	10.4	2705	40,001	6.8	6.5	7.0
Female	1626	17,683	9.2	8.6	9.6	2746	46,113	6.0	5.7	6.2
**Residential area**										
Capital (Seoul)	505	6153	8.2	7.5	9.0	865	16,129	5.4	5.0	5.7
Metropolitan	771	7427	10.4	9.7	11.1	1291	19,171	6.7	6.4	7.1
Rural	1890	19,689	9.6	9.2	10.0	3295	50,814	6.5	6.3	6.7
**Hypertension**										
No	827	10,529	7.9	7.3	8.4	1398	27,829	5.0	4.8	5.3
Yes	2339	22,740	10.3	9.9	10.7	4053	58,285	7.0	6.7	7.2
**Diabetes**										
No	2349	25,148	9.3	9.0	9.7	4000	66,182	6.0	5.9	6.2
Yes	817	8121	10.1	9.4	10.8	1451	19,932	7.3	6.9	7.7
**Hypercholesterolemia**										
No	2486	23,441	10.6	10.2	11.0	4312	61,548	7.0	6.8	7.2
Yes	680	9828	6.9	6.4	7.5	1139	24,566	4.6	4.4	4.9
**Atrial fibrillation**										
No	3096	33,017	9.4	9.1	9.7	5370	85,728	6.3	6.1	6.4
Yes	70	253	27.7	21.9	35.0	81	386	21.0	16.9	26.1
**Ischemic Heart Diseases**										
No	2938	32,018	9.2	8.9	9.5	5109	82,998	6.2	6.0	6.3
Yes	228	1251	18.2	16.0	20.8	342	3116	11.0	9.9	12.2
**Disabled due to cerebral lesions**										
No	2769	29,267	9.5	9.1	9.8	4754	76,024	6.3	6.1	6.4
Yes	397	4002	9.9	9.0	10.9	697	10,090	6.9	6.4	7.4
**Hospital characteristics**										
**CT**										
No	409	6052	6.8	6.1	7.4	782	15,695	5.0	4.6	5.3
Yes	2,757	27,217	10.1	9.8	10.5	4669	70,419	6.6	6.4	6.8
**MRI**										
No	715	8683	8.2	7.7	8.9	1329	22,523	5.9	5.6	6.2
Yes	2,451	24,586	10.0	9.6	10.4	4122	63,591	6.5	6.3	6.7
**Hierachy of hospital**										
General hospital	2,128	20,526	10.4	9.9	10.8	3500	53,201	6.6	6.4	6.8
Hospital	737	5193	14.2	13.2	15.3	1267	13,016	9.7	9.2	10.3
Clinics	301	7551	4.0	3.6	4.5	684	19,896	3.4	3.2	3.7
**TOTAL**	3,166	33,269	9.5	9.2	9.9	5451	86,114	6.3	6.2	6.5

CI, confidence interval; CT, computed tomography; MRI, magnetic resonance imaging; PY, person-years.

eTable 2. The seperate individual and regional hazard ratios and interactions

	Total				Capital				Metropolitan				Rural			
	**HR**	**95% CI**		**p-value**	**HR**	**95% CI**		**p-value**	**HR**	**95% CI**		**p-value**	**HR**	**95% CI**		**p-value**
**For 12-month mortality**																
**Individual income**																
Low	1.34	1.03	1.76	0.030	1.26	0.57	2.79	0.572	1.01	0.55	1.86	0.972	1.66	1.19	2.30	0.003
Middle	1.34	1.15	1.57	<.0001	1.28	0.83	1.97	0.259	1.20	0.87	1.67	0.271	1.47	1.21	1.78	<.0001
High	1.00				1.00				1.00				1.00			
**Neighborhood deprivation**																
Advantaged	1.00				1.00				1.00				1.00			
Disadvantaged	0.99	0.87	1.13	0.886	1.29	0.88	1.90	0.187	1.09	0.83	1.44	0.533	0.88	0.75	1.03	0.105
**Interaction term**																
	1.04	0.95	1.13	0.389	0.94	0.74	1.18	0.567	0.98	0.82	1.18	0.850	**1.13**	1.01	1.26	0.031
**For 36-month mortality**																
**Individual income**																
Low	1.43	1.17	1.75	0.001	1.43	0.79	2.58	0.236	1.18	0.75	1.86	0.475	1.68	1.31	2.16	<.0001
Middle	1.31	1.17	1.47	<.0001	1.32	0.96	1.83	0.089	1.19	0.93	1.53	0.162	1.40	1.21	1.63	<.0001
High	1.00				1.00				1.00				1.00			
**Neighborhood deprivation**																
Advantaged	1.00				1.00				1.00				1.00			
Disadvantaged	0.98	0.89	1.09	0.751	1.11	0.83	1.49	0.468	1.05	0.86	1.29	0.640	0.91	0.81	1.03	0.140
**Interaction term**																
	1.04	0.97	1.11	0.264	0.99	0.83	1.18	0.912	0.98	0.86	1.12	0.784	**1.11**	1.02	1.20	0.015

CI, confidence interval.

*Age group, sex, residential area, medical history, CT presence, MRI presence, hospital level, the number of beds, and the number of doctors were adjusted.

eFigure 1. Flowchart for sample selection (12-month overall mortality)

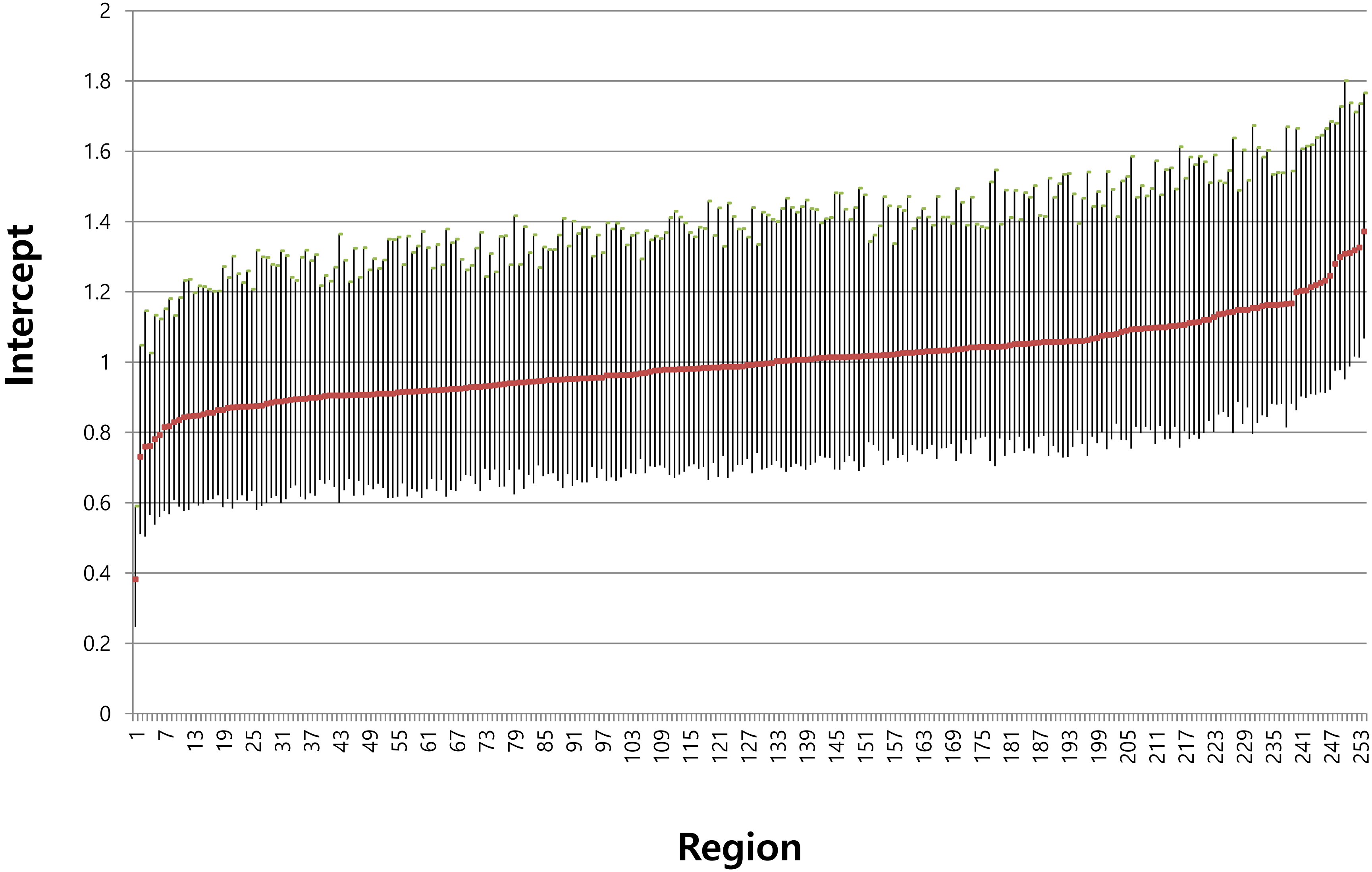

eFigure 2. Flowchart for sample selection (36-month overall mortality)

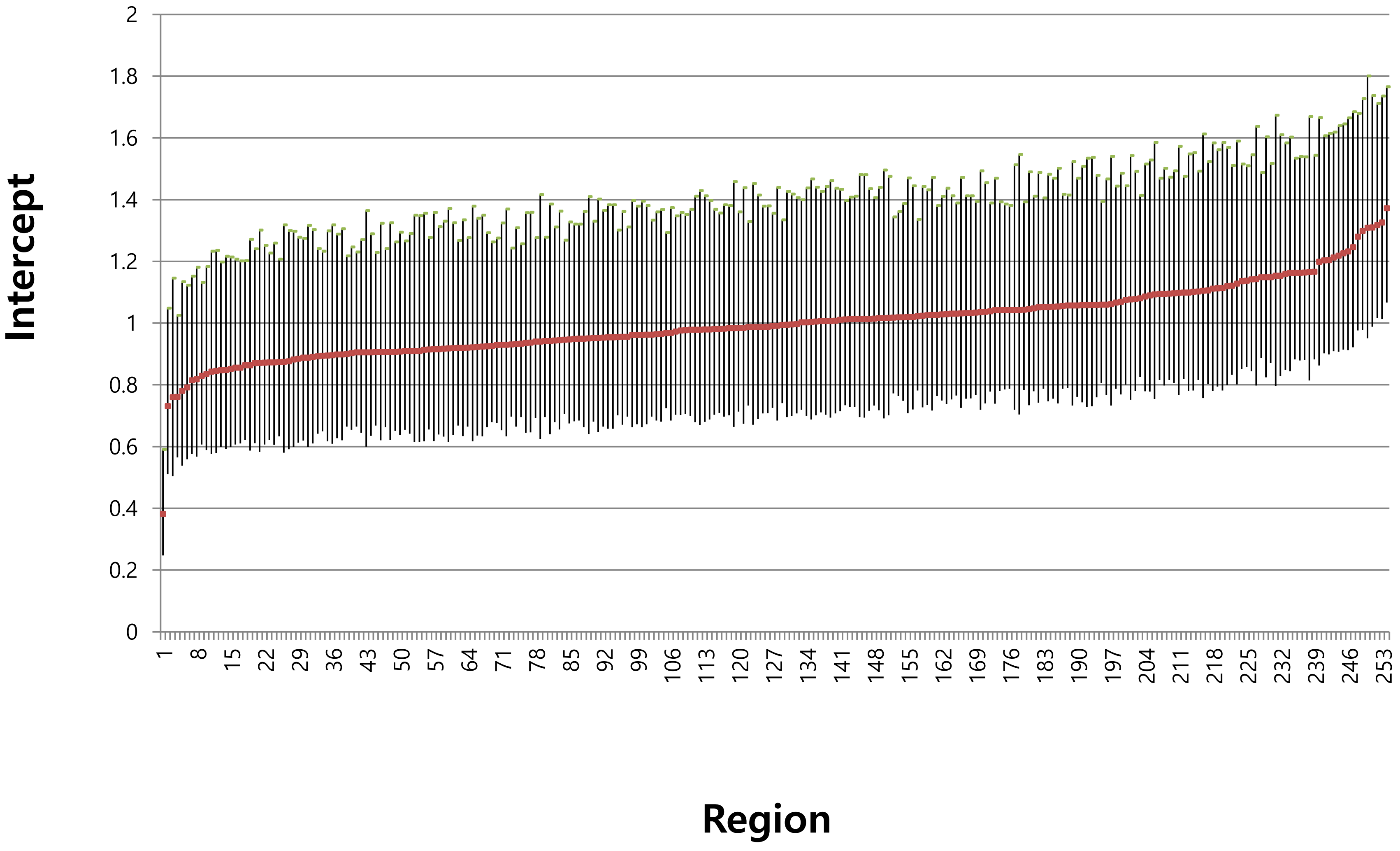

Supplement: Supplementary file 1 [file mmc1.pdf]
